# Semi-quantitative versus quantitative assessments of late gadolinium enhancement extent for predicting spontaneous ventricular tachyarrhythmia events in patients with hypertrophic cardiomyopathy

**DOI:** 10.1038/s41598-020-59804-8

**Published:** 2020-02-19

**Authors:** Young Jun Park, Seung-Jung Park, Eun-Kyung Kim, Kyoung Min Park, Sang-Cheol Lee, Young Keun On, June Soo Kim

**Affiliations:** Department of Medicine, Heart Vascular and Stroke Institute, Samsung Medical Center, Sungkyunkwan University School of Medicine, Seoul, Republic of Korea

**Keywords:** Cardiology, Cardiomyopathies

## Abstract

Extent of late gadolinium enhancement (LGE) quantified by cardiac magnetic resonance was reportedly helpful for predicting the risk of ventricular tachyarrhythmias (VTA) in hypertrophic cardiomyopathy (HCM) patients. However, only a few data exist on the clinical implication of semi-quantitative assessment LGE extent in this patient population. The extent of left ventricular (LV) LGE was measured in 310 consecutive HCM patients using semi-quantitative (summing the LV segments with LGE, spatial extent) and quantitative (calculating the LGE volume percentage [vol% of LGE] against the total LV myocardial volume) methods, respectively. LV LGE was detected in 255 (82%) patients, most frequently in the mid-LV septum (n = 160, 52%). During the 49 ± 45 month follow-up, spontaneous VTA events were observed in 48 patients (16%) including aborted sudden cardiac death (SCD), appropriate defibrillator shock, and non-sustained VTA. The extent of LGE assessed by the two different methods showed a strong positive correlation (Spearman’s r = 0.63, *P* < 0.001). Moreover, there was a graded increase in the rates of VTA with the LGE extent evaluated semi-quantitatively and quantitatively. The extent of LGE was identified as an independent predictor of VTA events and more extensive LGE (positive ≥ 4 segments) significantly raised the risk of VTA, irrespective of the presence of conventional risk factors for SCD including family history, unexplained syncope, LV wall thickness ≥30 mm. The extent of LGE, whether assessed by semi-quantitative or quantitative methods, was closely associated with an increased risk of spontaneous VTA events in HCM patients.

## Introduction

Late gadolinium enhancement (LGE) detected by cardiac magnetic resonance (CMR) imaging has been established as a useful method for *in vivo* detection of myocardial scarring in patients with ischemic and non-ischemic cardiomyopathies^1–3^. Patients with LGE compared to those without were found to be at greater risk of adverse outcomes including sudden cardiac death (SCD), ventricular tachyarrhythmias (VTA), appropriate shock of implantable cardioverter-defibrillator (ICD), and heart failure (HF)-related hospitalization^4^. In patients with hypertrophic cardiomyopathy (HCM) as well, the presence of LGE was identified as an independent risk factor for SCD or VTA^5,6^. Recently, extent of LGE was reported to be a better predictor of VTA/SCA for the HCM patients rather than the mere qualitative characterization (presence or absence) of LGE^7–9^. However, quantitative assessment of LGE is not always easy, frequently requiring a specialized software, longer time, and more cost for the measurement. Moreover, quantitative assessment of LGE has not been standardized. Semi-quantitative assessment of LGE, if well-correlated with quantitative one, would be easier to perform and very useful in usual clinical practices as was shown in patients with myocarditis^10^.

Previously, we reported that semi-quantitative measurement of LGE extent, counting the number of ventricular segments with LGE, was closely related to composite adverse events including atrial and ventricular arrhythmias, HF-related hospitalization, and stroke in the HCM patients^11^. However, limited data exist showing a good correlation between the extents of LGE measured by quantitative and semi-quantitative methods, particularly in terms of risk stratification for VTA/SCD. Therefore, in the present study, we evaluated the risks of VTA/SCD according to the extent of LGE assessed using two different methods; quantitative and semi-quantitative. We also investigated whether a more extensive LGE reflected a greater risk for ventricular arrhythmic events in this patient population.

## Methods

### Patient population

In all patients undergoing LGE-CMR at our institute, various parameters are prospectively collected and entered into our database such as clinical, electrocardiographic, and echocardiographic variables. In particular, information regarding previous syncopal episodes, family history of SCD, and unexplained palpitation are carefully included into the clinical data.

For the present study, we retrospectively selected a total of 310 consecutive patients who satisfied all the following criteria: (a) patients under regular follow-up in the cardiology clinic; (b) patients with an unexplained increase in end-diastolic left ventricular (LV) wall thickness (≥15 mm or ≥13 mm with positive family history of HCM) noted on echocardiographic examination (Vivid 7, GE Medical System, Milwaukee, WI or Acuson 512, Siemens Medical Solution, Mountain View, CA, USA); and (c) patients who underwent LGE-CMR examination between June 2008 and December 2011.

We excluded patients with (a) uncontrolled hypertension (systolic ≥160 or diastolic blood pressure ≥100 mm Hg despite use of antihypertensive drugs), (b) moderate or severe aortic valve stenoinsufficiency, (c) myocardial infiltrative or storage disease, (d) history of septal myectomy or alcohol ablation, (e) CMR study performed without LGE protocol, or (f) inadequate image quality for assessing the presence of LGE. The study protocol was approved by the institutional review board of our institute, and the requirement for written informed consent was waived.

### LGE-CMR protocol

The detailed LGE-CMR protocol used in the present study has been described in previous studies^12,13^. Briefly, all images were acquired using a 1.5-T scanner (Achieva, Philips Medical Systems, Best, Netherlands) with a SENSE cardiac coil. The LV endocardial and epicardial borders were planimetered using the short-axis images acquired at end-diastole and end-systole during the breath-holding period. The LV end-diastolic volume (EDV), end-systolic volume (ESV), myocardial volume, and ejection fraction (EF) were computed using Simpson’s algorithm. The LV myocardial mass was multiplied by the specific gravity of the myocardium (1.05 g/mol) to obtain the LV mass. The LV volume and mass data were then indexed for body surface area. Maximal LV wall thickness (LVWT) measured at end-diastole was also obtained across all short axis images.

The LGE was measured 10 minutes after administering intravenously 0.15 mmol/kg of gadolinium-diethylenetriamine pentaacetic acid (Magnevist, Bayer Schering Pharma, Berlin, Germany) in 10−12 contiguous slices. Slice thickness and interslice gap were 6 and 4 mm, respectively. A multi-shot turbo field echo breath-hold sequence with a non-selective inversion was used; typical repetition time of 4.6msec, time to echo of 1.4msec. We used a Look-Locker sequence to find an inversion time when healthy myocardium was optimally nulled, which was between 200 to 300 msec. The field-of-view and image matrix were 35 cm × 35 cm and 256 × 256, respectively.

### LGE-CMR analysis and quantification of its extent

CMR image analysis was performed at our CMR core laboratory by two experienced investigators (training level III) blinded to clinical information. The degree of the LV LGE was assessed using two different methods. First, the extent of LGE in spatial distribution was calculated semi-quantitatively by counting the number of LV segments showing visually-determined LGE. For this, the LV wall was divided into 13 segments using a similar method previously described: anterior, septal, inferior, and lateral walls in each basal, mid, and apical LV segments with the final apical-cap segment included^11,14^. Therefore, the semi-quantitative spatial extent of LV LGE scored from 0 to 13. Second, after finishing the semi-quantitative visual assessment, the LV LGE volume was quantified as a percentage of the total LV myocardial volume (vol%), which was computed by summing the percent area of LGE in each short-axis scan image, multiplied by the slice thickness along the entire LV. LGE was defined by the presence of enhanced areas with signal intensity greater than 6 standard deviation (SD) above that of remote normal myocardium^15^, which was automatically measured using commercialized software (CAAS MRV version 1.0, Pie Medical Imaging B.V, the Netherlands) and manually corrected as needed. Low intra- and interobserver variability in assessing LGE in our laboratory has been demonstrated in previous reports^3,12,13,15^.

### Clinical outcomes

The occurrence of VTA was investigated following the LGE-CMR examinations. Regular clinical and electrocardiographic follow-up (at 3 to 6 month intervals) was performed in all patients. If patients reported symptoms suggesting arrhythmia at any time, 12-lead ECG and 24-hour Holter monitoring were repeated. VTA events included SCD, ventricular fibrillation (VF), sustained ventricular tachycardia (≥100 beats/min for ≥30 seconds), hemodynamically unstable VTA requiring immediate cardioversion, VTA events terminated by appropriate ICD shock or anti-tachycardia pacing therapy, and non-sustained ventricular tachycardia (NSVT; ≥3 consecutive ventricular beats at a rate of ≥100 beats/min for <30 seconds). Only spontaneous VTA episodes with ECG documentation were included. However, induced VTA during electrophysiologic studies were excluded from the present analysis.

### Statistical analysis

Continuous variables were represented as medians with interquartile range (IQR) and categorical variables as number with percentages. Continuous variables were compared using the Mann-Whitney test whereas categorical variables using the Fisher’s exact test. Correlations (1) between the extents of LGE measured by two different methods, and (2) between the (quantitative and semi-quantitative) LGE extent and the rate of VTA were assessed by the Spearman rank correlation coefficient, respectively. VTA-free survival rates according to the degree of LV LGE were analyzed using Kaplan-Meier curves and differences between survival rates were assessed using the log-rank test. Cox regression analyses were performed to determine independent predictors for the development of VTA, after testing the proportional hazards assumption based on Schoenfeld residuals. Statistically significant variables identified from univariate analysis along with other clinical variables considered important irrespective of their univariate P-values, were included in multivariate analysis model. All P-values were two-sided, and a P-value < 0.05 was considered statistically significant. All data were analyzed using PASW Statistics 23 software for Microsoft (SPSS Inc., Chicago, IL, USA).

### Ethics statement

The Institutional Review Board (IRB) or our institute approved the study protocol (IRB No. 2019-09-133). The present study was carried out in accordance with the ethical principles of the Declaration of Helsinki and the requirement for written informed consent was waived.

## Results

### Patient demographics

The median (IQR) age of the 310 patients was 55 (49–65) years and the proportion of male patients was 78% (241/310). A family history of SCD was noted in 12% of patients (37/310). Twenty-eight patients (9%) had unexplained syncopal episodes. The median (IQR) values of LV EF and the maximal LVWT were 66 (61–71) % and 18 (15–23) mm. Patients with apical, asymmetric septal, and mixed/diffuse HCM subtypes accounted for 35, 41, and 24% of the total population, respectively.

### The distribution and extent of LV LGE

**Figure 1:**
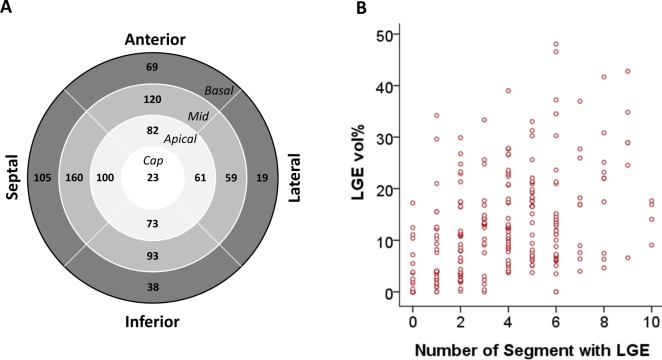
Distribution and extent of the LV LGE. The number of patients with LGE in each LV segment was presented (**A**) with the mid-LV level septal segments enhanced most frequently by gadolinium (n = 160). There was a strong positive correlation between the extents assessed by semi-quantitative (number of LV segments with LGE) and quantitative (LGE vol%) methods (**B**). LV, left ventricle; LGE, late gadolinium enhancement; LGE vol%, LGE volume percent.

Ventricular LGE was detected in 82% of patients (255/310) who underwent the LGE-CMR study. In the LV short-axis images, LGE was detected primarily in the septal wall, followed by the anterior, inferior, and lateral walls in descending order of frequency. Along the LV long-axis, the mid-LV segments displayed LGE more frequently than the apical or basal ones. Therefore, the mid-LV septal segment was the most common site of gadolinium enhancement. A detailed spatial distribution of the LV LGE is presented in Fig. 1A.

The extent of LGE was quite variable among individuals in semi-quantitative spatial distribution (median score 3, range 0 to 10), as well as in quantitative volume percentage (median 9.4%, range 0 to 48%). However, the extents of LGE, calculated by the two different methods, demonstrated a good positive correlation (r = 0.63, *P* < 0.001, Fig. 1B).

### Comparison of patients with and without LV LGE

Patients with LGE were younger than those without (*P* = 0.029). There was a trend toward a higher frequency in the family history of SCD, HF, previous stroke, and beta-blocker use in the LGE group compared to the no LGE group (Table 1).

**Table 1:** Comparison of baseline characteristics.

	LGE – (n = 55)	LGE + (n = 255)	*p* Value
***Demographic variables***			
Age (years)	62 (55–74)	57 (50–66)	0.029
Male gender, n (%)	40 (73)	200 (78)	0.376
Family history of SCD, n (%)	4 (7)	33 (13)	0.162
Previous history of syncope, n (%)	5 (9)	23 (9)	1.000
Diabetes, n (%)	7 (13)	31 (12)	1.000
Hypertension, n (%)	21 (38)	113 (45)	0.454
Congestive heart failure, n (%)	6 (11)	46 (18)	0.237
Atrial fibrillation, n (%)	3 (5)	18 (7)	1.000
Stroke, n (%)	3 (5)	25 (10)	0.438
***Medication***			
Amiodarone, n (%)	2 (4)	4 (2)	0.289
Beta-blocker, n (%)	28 (51)	160 (63)	0.128
ACEI or ARB, n (%)	14 (26)	84 (33)	0.338
Diuretics, n (%)	3 (5)	23 (9)	0.591

ACEI, angiotensin-converting enzyme inhibitor; ARB, angiotensin receptor blockers; LGE, late gadolinium enhancement; SCD, sudden cardiac death.

LV EDVi and ESVi did not differ significantly between the two groups (Table 2). However, patients with LGE had much greater LV mass index (*P* < 0.001) and maximal LVWT (*P* = 0.001) than those without LGE. Additionally, the QRS duration (*P* = 0.006) and corrected QT interval (*P* = 0.037) were significantly longer in patients with LGE.

**Table 2:** Comparison of electrocardiographic and echocardiographic variables.

	LGE –(n = 55)	LGE + (n = 255)	*p* Value
***CMR-LGE variables***			
LV EDV index, mL/m^2^	70 (61–84)	76 (66–86)	0.212
LV ESV index, mL/m^2^	22 (17–28)	21 (17–28)	0.926
LV EF, %	69 (64–74)	71 (62–76)	0.665
Maximal LV wall thickness, mm	15 (13–18)	17 (104–155)	0.001
Maximal thickness ≥30 mm, n (%)	6 (11)	22 (9)	0.605
LV Mass index, g/m^2^	67 (53–92)	91 (76–114)	<0.001
***Electrocardiographic variables***			
QRS duration, msec.	89 (84–96)	94 (87–103)	0.006
QTc interval, msec.	435 (423–448)	446 (427–470)	0.037

CMR, cardiac magnetic resonance; EDVi, end diastolic volume index; EF, ejection fraction; ESVi, end systolic volume index; LGE, late gadolinium enhancement, LV, left ventricular; QTc, corrected QT.

### Associations between ventricular arrhythmias and the LV LGE extent assessed by two different methods

In the mean follow-up duration of 49 ± 45 months, 48 (16%) patients developed VTA events: aborted SCD due to VF (n = 2), appropriate ICD shock for VF (n = 4), and NSVT (n = 42). There were only two cases of cancer-related deaths, and no cardiac deaths during the follow-up period.

**Figure 2:**
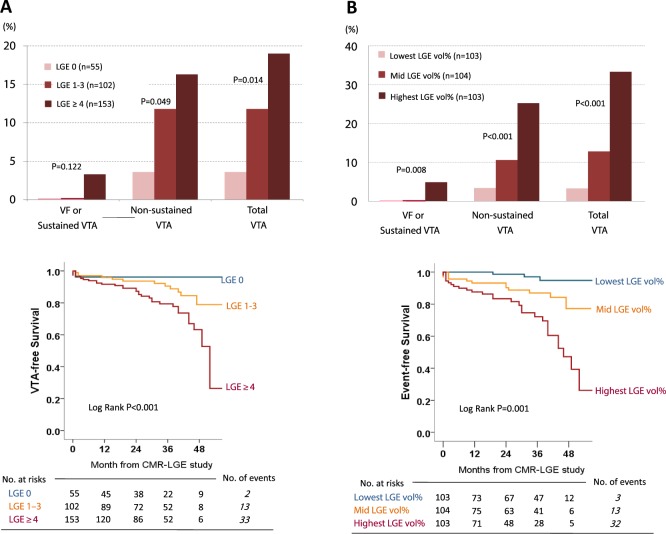
Risk of ventricular tachycardia depending on the extent of LV LGE. There was a graded increase in the rate of ventricular tachyarrhythmic events with the degree of LV LGE whether it was measured semi-quantitatively (**A**) or quantitatively (**B**). Event-free survival also declined as a function of the extent of LGE (**C,D**). LGE 0, LGE 1–3, and LGE ≥4 refer to the patient groups that showed LGE in 0, 1–3, and ≥4 LV segments, respectively. Lowest, Mid, and Highest LGE vol% refer to the patient groups divided by the level of LGE volume percentage; the lowest (<5.3%; n = 103), mid (5.3 to 17.6%; n = 104), and the highest tertile (≥17.6%; n = 104) groups. LGE, late gadolinium enhancement; VF, ventricular fibrillation; VTA, ventricular tachyarrhythmia.

To assess the rate of VTA depending on the semi-quantitative extent of LV LGE, we divided the 310 patients into three groups; Supramedian-LGE (≥4 segments with LGE; n = 153), Intermediate (Inframedian)-LGE (1 to 3 segments with LGE; n = 102), and No-LGE (no segment with LGE, n = 55) groups. With increase in the semi-quantitative (spatial) extent of LGE, there was a graded elevation in the rates of NSVT (P = 0.049) and total VTA (P = 0.014, Fig. 2A). Additionally, more serious events such as VF and sustained VTA were not documented in the 2 subgroups with LGE < 4 segments. Interestingly, similar trends were reproduced even when patients were re-categorized into three groups according to the quantitative extent of LGE (LGE vol%); the Lowest (<5.3%; n = 103), Mid (5.3 to 17.6%; n = 104), and the Highest tertile (≥17.6%; n = 103) groups. The Highest tertile group in the LGE vol% demonstrated the worst prognosis, whereas the Lowest tertile group revealed the most benign course (Fig. 2B).Unadjusted Kaplan-Meier survival curves depending on the semi-quantitative and quantitative extent of LGE are depicted in Fig. 2C,D. Supplemental Figure shows representative cases with the different risks for VTA according to the different burden of LV LGE.

**Figure 3:**
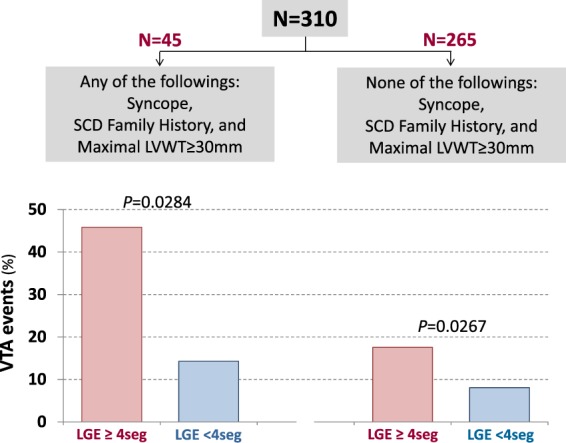
Risk of ventricular tachyarrhythmia in the subgroups sorted by the presence of conventional risk factors for sudden cardiac death. The presence of more extensive LGE made the risk of ventricular arrhythmia more striking irrespective of the presence of conventional risk factors for SCD. LGE, late gadolinium enhancement; LVWT, left ventricular wall thickness; SCD, sudden cardiac death.

The degree of LV LGE, if evaluated as the spatial extent or LGE vol% in Cox regression analysis, was independently associated with the development of VTA, even after adjustment for potential confounders such as age, HF history, hypertension, QRS duration, baseline LV EF, maximal LVWT, LV EDVi, and LV mass index (Table 3). In addition, LGE, if treated as a continuous or dichotomized variable, maintained an independent association with VTA (Table 3). Moreover, the presence of more extensive LV LGE (≥4 segments) strikingly elevated the risk of VTA in patients with and without other risk factors for SCD (Fig. 3).

**Table 3:** Risk of ventricular arrhythmia associated with LGE extent.

Variables	Hazard Ratio^*^ (95% C.I.)	*p* Value
***In semi-quantitative extent of LGE***		
**Supramedian LGE**^†^ (≥4seg) vs. Inframedian LGE	3.55 (1.68–7.49)	0.001
**Supramedian LGE**^†^ vs. LGE–	5.21 (1.32–39.0)	<0.001
**LGE extent**^‡^ (number of segments with LGE; 0~13)	1.25 (1.09–1.43)	0.001
***In quantitative extent of LGE***		
**Supramedian vol%**^†^ vs. inframedian vol%	2.25 (1.06–4.74)	0.034
**Highest tertile vol%**^†^ vs. Lowest tertile vol%	8.31 (2.26–30.5)	0.001
**LGE vol%**^‡^ (in the LV myocardial volume; 0~100%)	1.07 (1.03–1.11)	<0.001

*Adjusted with age, heart failure history, hypertension, QRS duration, baseline LV EF, maximal LV wall thickness, LV EDVi, and LV mass index.

†Indicates LGE was treated as a dichotomous variable in multivariable analyses.

^‡^Indicates LGE was treated as a continuous variable in multivariable analyses.

LGE, late gadolinium enhancement; LGE vol%, LGE volume percent; LV, left ventricle.

## Discussion

### Main findings

The present study reveals a significant positive correlation between the extents of LV LGE assessed by two different methods; semi-quantitative (number of LV segments with LGE) and quantitative (LV LGE vol%) ones. In addition, there was a graded increase in the rate of VTA events with the extent of LV LGE, if assessed semi-quantitatively or quantitatively, in patients with HCM. Moreover, the presence of more extensive LV LGE (≥4 segments) made the risk of VTA more striking irrespective of conventional risk factors for SCD such as family history of SCD, unexplained syncopal episode, or maximal LVWT ≥30 mm (Fig. 3).

Although there have been several studies investigating arrhythmic risk as a function of LGE extent in this patient population^6–13,16^, the present study is the first, to our knowledge, to show a strong positive correlation between the LGE extent assessed by the two different ways; counting the number of LV segments showing LGE and calculating the LGE volume as a percentage of the total LV myocardial volume.

### Extent of LGE and risk of ventricular tachyarrhythmias

The risk of VTA was highest in patients with the greatest degree of LV LGE, regardless of whether LGE extent was evaluated semi-quantitatively (LGE positive ≥4 segments) or quantitatively (LGE vol% ≥17.6%). Interestingly, our findings are in good agreement with those from previous studies in which the arrhythmic risk was significantly elevated in patients with LGE volume ≥15%^7,8^ or LGE ≥4 segments^17^. We also found that the LV LGE, if treated as a continuous or dichotomized variable, maintained an independent association with risk of VTA in various multivariate analysis models that were adjusted for potential confounding risk factors.

It has been well-demonstrated that the presence of LGE on CMR imaging reflects the presence of myocardial scarring or peri-infarct zones in patients with ischemic or non-ischemic cardiomyopathy^1,2^. As the myocardium is replaced by scar tissue following any disease process, ventricular activation and myocardial conduction becomes more heterogeneous and delayed around the scar or peri-infarcted zone, thereby increasing the risk of reentrant fatal ventricular arrhythmia. In a cross-sectional study that included 108 patients with HCM, the QRS complex on 12-lead ECG became more fragmented along with the extent of LV LGE^18^. In our data, the extent of LGE (as a continuous variable) revealed significant positive correlations with QRS duration (in vol%, r = 0.176, *P* = 0.004; in spatial extent, r = 0.192, *P* = 0.001). These findings might suggest a greater conduction delay in HCM patients with a higher LGE burden. Additionally, more frequent NSVT events detected in patient with more extensive LGE, could serve as triggers for sustained VTA events.

### Clinical implications

ICD implantation is the most effective treatment for the prevention of SCD, which is the leading cause of death in HCM patients^19,20^. However, the annual rates of appropriate ICD therapy have been reportedly low (1 to 10%), even if patients were selected for ICD treatment based on conventional risk factors^20^. In a multicenter ICD registry study that included 506 HCM patients, the likelihood of appropriate discharge was not increased by number of conventional risk factors, suggesting an ongoing need for another marker that can improve current risk-stratification strategies^19^. Scar extent quantified by LGE-CMR is being actively investigated as a promising biomarker in this respect. Several studies have already proved that quantitative assessment of LGE was better than qualitative one for risk stratification of VTA events in HCM patients^7–9^. However, a specialized software for LGE quantification is not always available in every imaging center. If available, scar quantification often depends on manual tracing of the hyperenhanced areas on LGE-CMR images, which is laborious, time-consuming, and operator-dependent, limiting the reproducibility of the method^16^.

Our data showed a semi-quantitative method (visual counting the number of LV segments with LGE) was comparable to quantitative one (vol% calculation) in estimating the extent of LV LGE and the risk of VTA events during the follow-up. There was a similar stepwise increase in the rate of VTA according to the extents of LV LGE determined by the two methods. Additionally, the risk of VTA events was significantly elevated in patients with more extensive LGE, regardless of the presence or absence of conventional risk factors (Fig. 3). Therefore, the semi-quantitative visual counting of the LV segments with LGE could be an easier and more practical initial way for estimating the LV LGE extent and the risk of VTA/SCD.

### Study limitations

The present study has the limitations inherent to a retrospective observation study, in which unmeasured confounders may preclude definite conclusions. Therefore, prospective studies on a larger scale are needed to validate our results in this patient population.

Second, ventricular arrhythmia was used as a surrogate marker for SCD in the present study, with NSVT comprising the majority of VTA events. However, most SCD cases are thought to be caused by VTA. In our study, aborted SCD or appropriated ICD therapy occurred only in the patient group with the greatest LGE burden. The risk of NSVT also gradually increased with the extent of LGE.

Next, we did not compare the performance of the two different methods to predict risk of other clinical outcomes such as new-onset HF or atrial fibrillation, stroke, or cardiac death. Therefore, further study might be worthwhile to ascertain whether visual grading is not inferior to quantitative method in various risk assessment.

Finally, quantitative assessment of CMR-LGE was not performed using different methods in the present study; it was reported that a relatively low-SD threshold (e.g., 2−3SD) can be better for capturing entire arrhythmic substrate in HCM patients with more diffuse fibrosis, compared to a higher SD (i.e., 5−7SD). In addition, the full width at half maximum (FWHM) technique is known to have the best intra- and inter reader variability, particularly in cardiac conditions with diffuse fibrosis^21^. However, there was a recent study which showed that CMR-LGE measured at 4−5SD yielded the closest approximation to the extent of total fibrosis measured by the histopathological standard of reference in HCM patients who underwent CMR imaging study and septal myectomy^2^. We also believe that a close correlation must have been maintained between our visual grading and quantitative assessment even if we had used a low-SD method.

## Conclusions

A strong positive correlation was observed in the extents of LV LGE assessed by quantitative and semi-quantitative methods for patients with HCM. Additionally, the degree of LV LGE, whether measured in terms of semi-quantitative spatial extent or quantitative volume percentage, was closely associated with an increased risk of spontaneous VTA events in this patient population.

## Supplementary information

Supplementary Data.
