# Supplementary material for: Semi-quantitative versus quantitative assessments of late gadolinium enhancement extent for predicting spontaneous ventricular tachyarrhythmia events in patients with hypertrophic cardiomyopathy
Source: Sci Rep. 2020 Feb 19;10:2920. doi: 10.1038/s41598-020-59804-8 (PMC7031259; doi:10.1038/s41598-020-59804-8)
Supplement: Supplementary file 1 — Supplementary Data. [file 41598_2020_59804_MOESM1_ESM.pptx]

## Slide 1
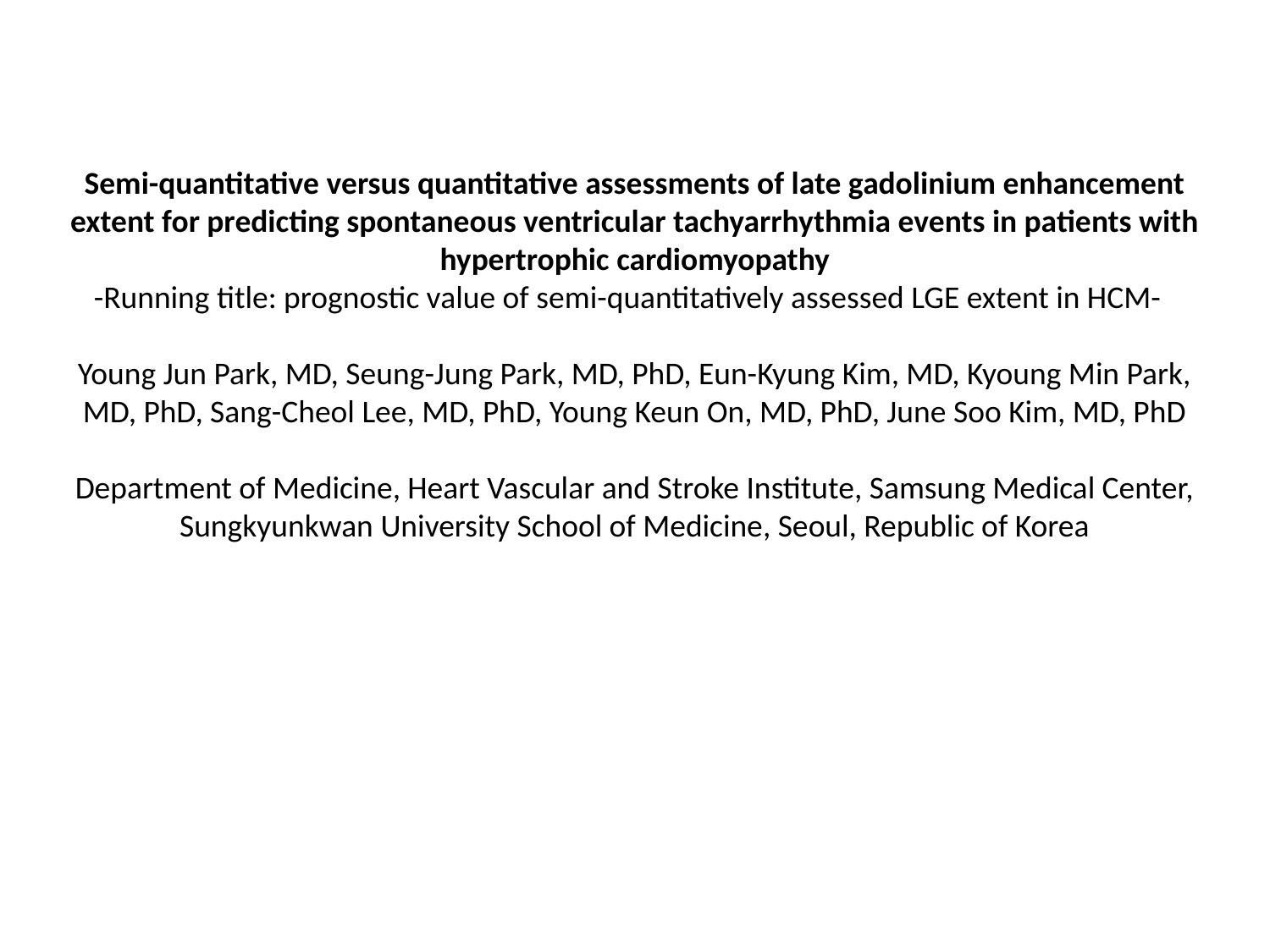

Semi-quantitative versus quantitative assessments of late gadolinium enhancement extent for predicting spontaneous ventricular tachyarrhythmia events in patients with hypertrophic cardiomyopathy
-Running title: prognostic value of semi-quantitatively assessed LGE extent in HCM-
Young Jun Park, MD, Seung-Jung Park, MD, PhD, Eun-Kyung Kim, MD, Kyoung Min Park, MD, PhD, Sang-Cheol Lee, MD, PhD, Young Keun On, MD, PhD, June Soo Kim, MD, PhD
Department of Medicine, Heart Vascular and Stroke Institute, Samsung Medical Center,
Sungkyunkwan University School of Medicine, Seoul, Republic of Korea

## Slide 2
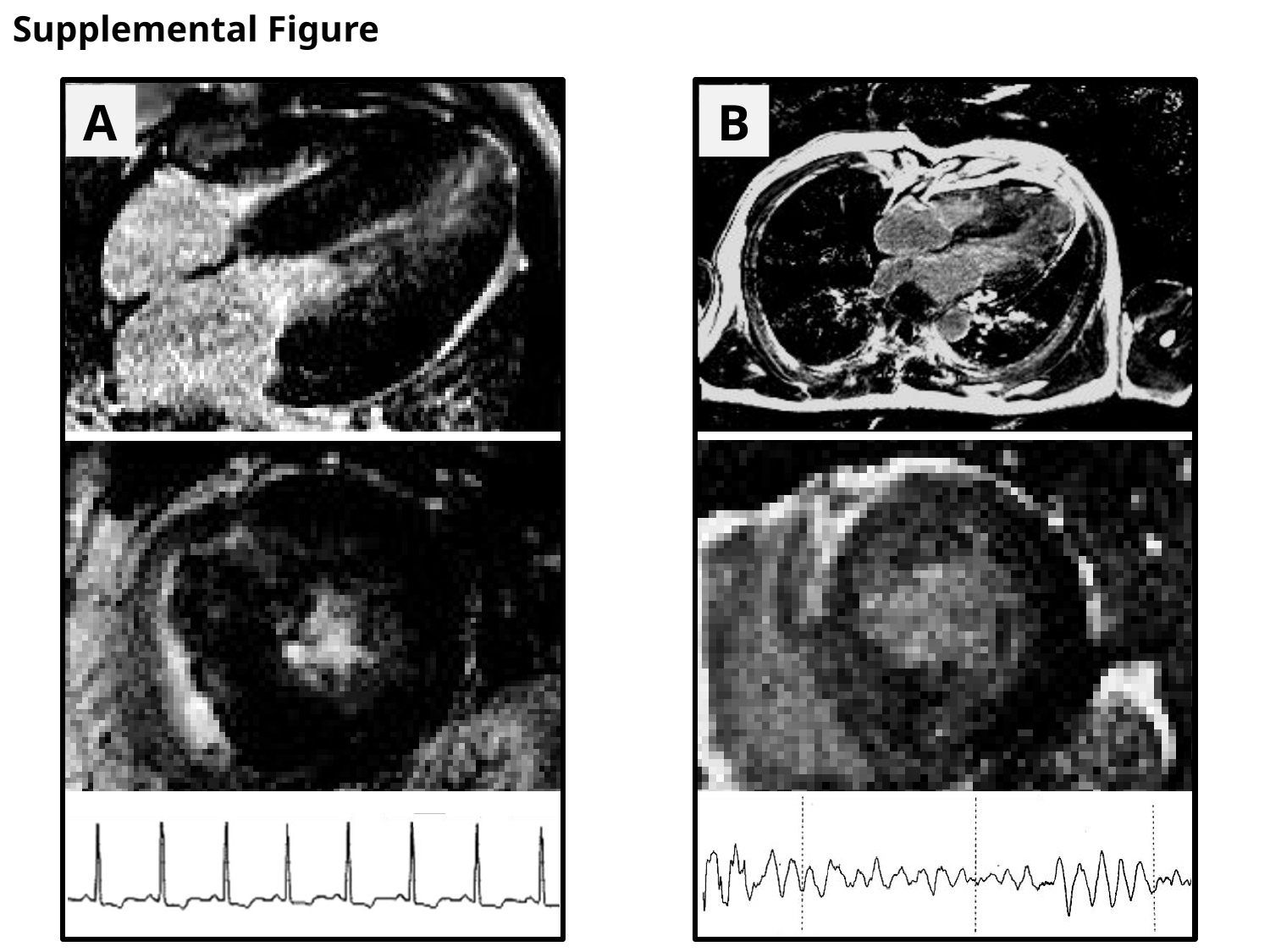

Supplemental Figure
A
B

## Slide 3
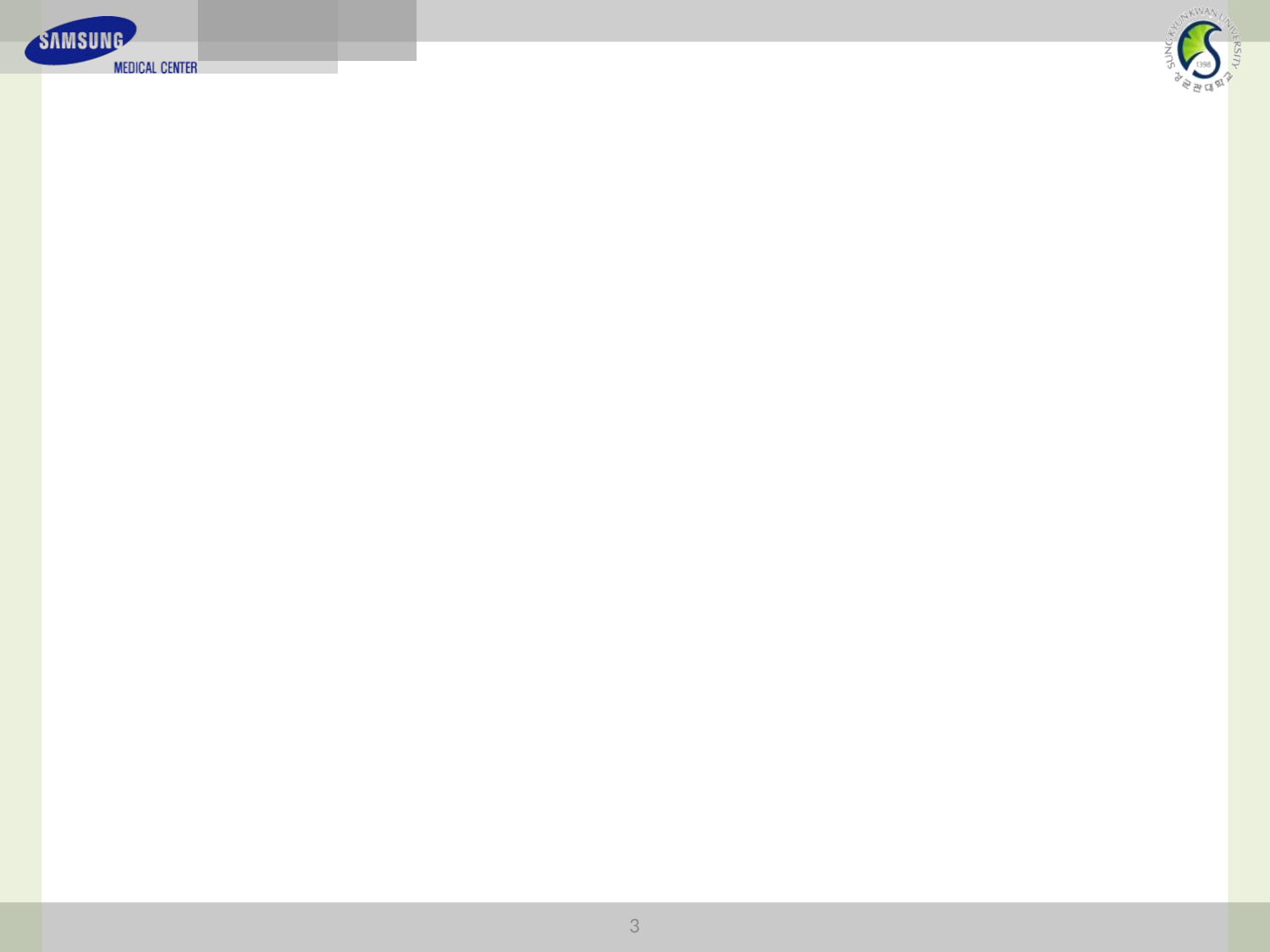

3
